# Laparoscopic versus Open Appendectomy: A Prospective Comparative Study and 4-Year Experience in a Tertiary Care Hospital

**DOI:** 10.1055/s-0042-1751112

**Published:** 2022-08-22

**Authors:** Aftab H. Shaikh, Amarjeet E. Tandur, Sachin Sholapur, Gajanan Vangal, Ajay H. Bhandarwar, Ahana Ghosh, Abhishek Rathod

**Affiliations:** 1 Department of General Surgery, Grant Government Medical College and JJ Group of Hospitals, Mumbai, Maharashtra, India; 2 Department of General Surgery, Civil Hospital, Ahmednagar, Gujarat, India

**Keywords:** laparoscopic, appendectomy, appendicitis, acute, recurrent

## Abstract

**Background** The aim of this study was to validate the pros of laparoscopic appendectomy (LA) over open appendectomy (OA) and to compare various primary outcome measures in the management of acute and recurrent appendicitis.

**Study Design** Prospective comparative study.

**Place and Duration** Between June 2015 and October 2019 in JJ Hospital, Mumbai.

**Materials and Methods** Total of 60 patients with acute and recurrent appendicitis were included in the study. Thirty patients underwent OA and 30 underwent LA. Both groups were comparable clinicopathologically and demographically. Various intraoperative and postoperative parameters were compared. Continuous variables were expressed as mean ± standard deviation and categorical variables were expressed as percentages. Mann–Whitney U test was used to compare continuous variables and chi-squared test was used to compare categorical variables. *p* -Value≤0.001 was considered to be statistically significant.

**Results** The median age of patients undergoing OA and LA was 24.9 and 25.2 years ( *p* = 0.221), respectively. Female: male ratio in OA and LA was 1.30 and 1.14, respectively ( *p* = 0.795). Mean operative duration in LA and OA group was 47.17 ± 14.39 minutes and 36.9 ± 12.33 minutes ( *p* = 0.001), respectively. Mean length of postoperative stay in LA and OA group was 3.69 ± 0.71 days and 5.28 ± 0.63 days ( *p* = 0.000), respectively. Median visual analogue scale score in LA and OA group was 3.5 and 5 ( *p* = 0.001), respectively. Mean time to return to normal activity in LA and OA group was 8.13 ± 1.33 days and 10.10 ± 2.20 days ( *p* = 0.000), respectively. About 6.66% patients in LA group and 13.33% in OA group had postoperative wound infection ( *p* = 0.652). Mean scar scale scoring done on 30th postoperative day was 4.23 in LA and 8.23 in OA ( *p* = 0.000).

**Discussion and Conclusion** LA is more promising than OA in the management of acute and recurrent appendicitis. LA offers lesser operative site pain in the postoperative period, shorter postoperative hospital stays, earlier recovery, and return to normal activities and cosmetically better scars on 30th day follow-up. No conversions or significant difference in wound related complications were seen in both groups. Prolonged intraoperative duration was the only drawback of LA.

Acute appendicitis is the most common cause of acute abdomen in almost all age groups. 1 2 Ever since Charles McBurney described traditional appendectomy in 1894 for acute appendicitis, open appendectomy (OA) flourished as gold standard treatment for appendicitis. 3 OA was considered safe, effective, and standard modality of treatment in appendicitis for almost a century. Though easy to perform, OA had a plethora of drawbacks due to variability in the inflammatory process and position of appendix, increased postoperative pain, prolonged hospital stays, delayed return to normal activities, wound- and scar-related complications, and inability to visualize the concomitant pathologies. With the advent of minimally invasive surgery (MIS), laparoscopic cholecystectomy gained immense popularity for the management of symptomatic gallstone disease; however, it was not the same case with laparoscopic appendectomy (LA).

Semm, a German gynecologist, performed the first LA in 1984. 4 With advancing MIS, the incidence of LA has increased in the past decade. LA offered lesser postoperative morbidity, early recovery, opportunity to perform a diagnostic laparoscopy, and cosmetically better scars than OA. 5 6 Though rapidly advancing surgical practice is more inclined toward MIS, the drawbacks associated with LA such as prolonged intraoperative duration, steep learning curve, higher incidence of intraabdominal abscess, and cost-ineffectiveness cannot be ignored. The relative pros and cons of OA and LA in the management of appendicitis have been debated and compared by numerous randomized controlled trials in the past; however, the dilemma in choosing a single best procedure in a clinical scenario is still lingering. 7

This prospective comparative study describes our experience and compares various primary outcome measures in the management of acute and recurrent appendicitis by OA and LA in a tertiary care hospital.

## Materials and Methods

This single-center prospective comparative study was conducted in the General Surgical Units of JJ Hospital in Mumbai, India, between June 2015 and October 2019. The objective of the study was to compare various intraoperative and postoperative factors influencing the management of acute and recurrent appendicitis by OA and LA. Primary outcome measures such as intraoperative duration, postoperative pain, length of postoperative hospital stay, time for returning to normal activity, postoperative complications, rate of conversion, and subjective cosmesis were the parameters considered for comparison. A total of 60 patients presenting to the surgical outpatient department with right lower quadrant pain were included in the study after obtaining informed consents from the subjects and a clearance from the Hospital Ethical Committee. Diagnosis was made after a thorough clinical examination, Alvarado MANTRELS scoring (score> 7), and/or ultrasound/contrast-enhanced computed tomography (CT) scan evidence of an inflamed appendix. The inclusion and exclusion criteria for the study were as follows:

### Inclusion Criteria

1. Patients presenting with right iliac fossa pain with diagnosis of acute and recurrent appendicitis after clinical exam, MANTRELS score (>7) and USG/CT scan.
2. Patients between the age of 12 and 70 years.
3. Nonpregnant patients.
4. Patients of American Society of Anesthesiologists (ASA) class 1 and 2.
5. Patients consenting for the procedure and ready to abide by the follow-up protocols.

### Exclusion Criteria

1. Patients below 12 years and above 70 years of age.
2. Cases with chronic appendicitis, phlegmon, and appendicular abscess.
3. Subjects not fit after preanesthetic check and ASA class ≥3.
4. Pregnant patients.
5. Subjects not willing to consent for the procedure and not feasible with regular follow-up.

Subjects were divided into two groups by lottery method into the ones undergoing LA and OA to avoid selection bias. Patients were admitted on the day of surgery; routine laboratory, and radiological tests including complete cell counts, liver and renal function tests, hepatitis B, C, and HIV screening, chest and abdominal radiographs, electrocardiogram, and ultrasonography of the abdomen and pelvis were performed. Patients were explained about the risks and benefits of both the procedures and written informed consents were obtained. Surgeries were performed in same operating room complex by five different surgeons with adequate similar skills in both open and laparoscopic surgeries. All patients received a dose of third-generation cephalosporin intravenous antibiotic at the time of induction.

**Fig. 1:**
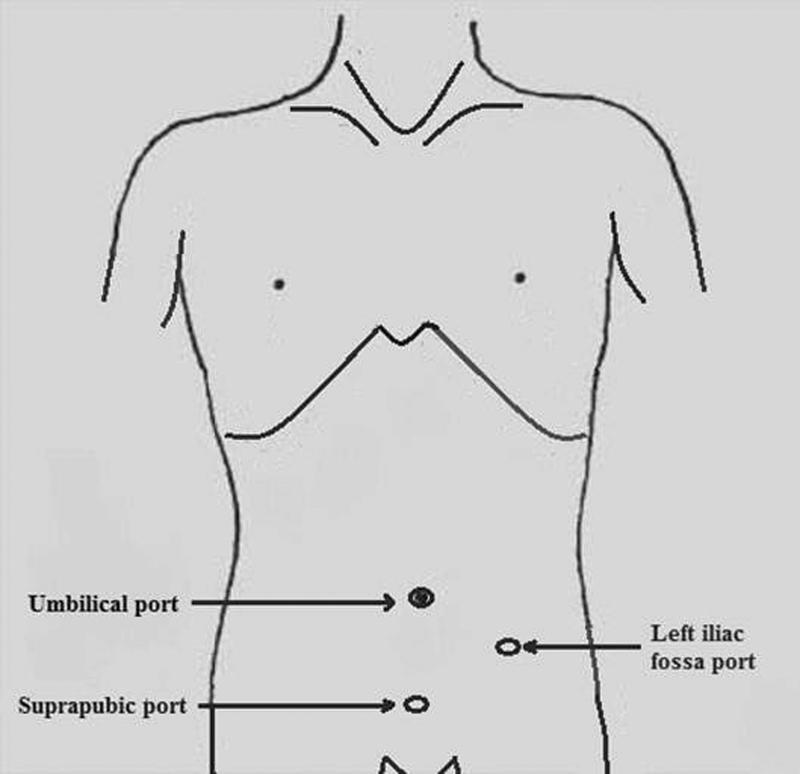
Port position in laparoscopic appendectomy.

OA was performed under spinal anesthesia with traditional Lanz incision. Muscles were spilt, peritoneum incised, appendix was mobilized, and mesoappendix was ligated with polyglactin 2–0 sutures and divided. Appendix was then crushed, transfixed, ligated at the base with polyglactin 2–0 and divided. Skin was closed with simple sutures using Nylon 3–0. LA was performed with conventional three-port technique. Umbilical port (10 mm) was inserted and pneumoperitoneum was created by open approach, followed by 5 mm ports in the suprapubic region and the left iliac fossa under vision. Mesoappendix was sealed using bipolar electrosurgical device and divided. Appendix was doubly ligated at the base with Roeder's knot using chromic catgut and was divided and retrieved in an endobag through the 10 mm port site. Skin was closed with simple sutures using Nylon 3–0. Lavages were given in two cases of OA and one case of LA. Drains were not inserted in any LA or OA case. Appendicitis was confirmed after histopathological examination of all the samples. Patients were started on oral liquids on postoperative day 1 and day 2, respectively, in LA and OA patients. Soft diet was started once oral liquids were tolerated well ( Figs. 1 2 3 ).

**Fig. 2:**
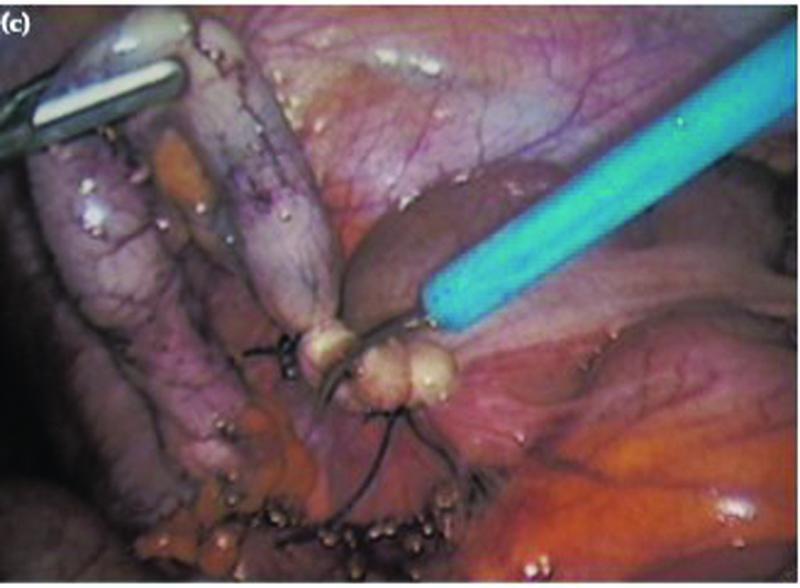
Appendix doubly ligated with laparoscopic Roeder's knot.

**Fig. 3:**
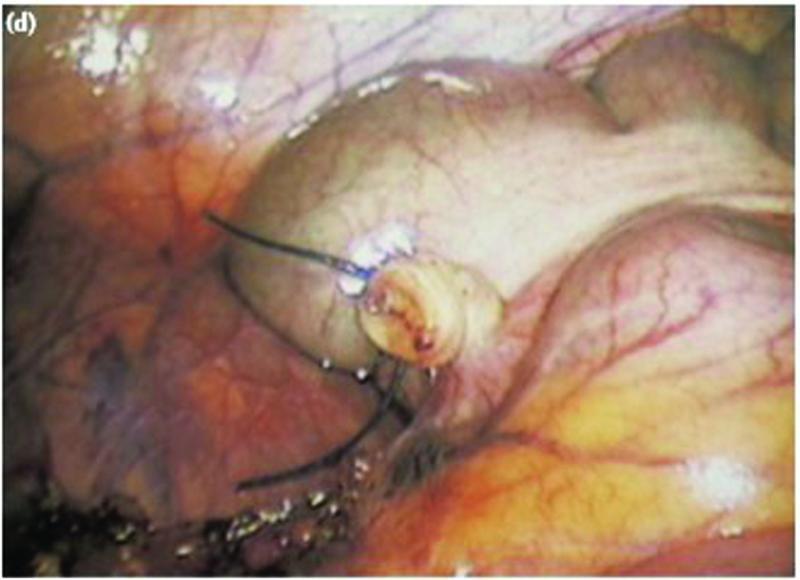
Appendicular stump after division.

Various intraoperative and postoperative parameters were recorded and analyzed. Intraoperative duration (in minutes), which was defined as the time from skin incision to the last stitch of skin closure in OA and from infraumbilical port insertion to closure of the last defect in LA, was recorded in all cases. Intraoperative complications such as hemorrhage, visceral injuries, and conversion to open surgery were recorded. Postoperative complications such as hemorrhage, wound discharge, wound gape, and intraabdominal abscess were looked for. Postoperative pain was assessed on 1st, 2nd, and 7th postoperative days using visual analogue scale (VAS). Length of postoperative stay defined as the number of nights spent in the hospital after surgery was noted in all cases. Time for returning to normal activities was defined as the time taken after surgery (in days) when abdominal discomfort did not interfere with normal daily activities. The final cosmesis in both LA and OA, as perceived by the patient using the Scar Scale on a scale of 3 to 15, with 3 being the best result and 15 being the worst, was recorded on 30th postoperative day. Patients were followed up at the end of 1st, 2nd, and 4th weeks after the surgery. Sutures were removed at the end of 1st postoperative week in all cases.

## Results

Total of 60 patients were included in the study. All patients undergoing appendectomy for acute and recurrent appendicitis were explained about the merits and demerits of OA and LA. Continuous variables such as age, operative duration, length of hospital stay, VAS score, and time to return to normal activity were expressed as mean ± standard deviation and categorical variables such as gender, conversion rates, and postoperative complications were expressed as percentages. Details were entered into the Statistical Package for the Social Sciences (SPSS) software for statistical analysis of the data. Mann–Whitney U test was used to compare continuous variables and chi-squared test was used to compare categorical variables. *p* -Value≤0.001 was considered to be statistically significant.

Out of 60 patients, 30 underwent OA and 30 underwent LA. Both the groups were comparable in their clinicopathological parameters and all efforts were made to avoid confounding factors. No mortality, readmission, or re exploration was encountered in either group. The median age of patients undergoing OA and LA was 24.9 and 25.2 years, respectively. *p* -Value was 0.221, indicating no statistically significant difference between the two groups with respect to age. The number of females in both groups was higher in comparison to males with female: male ratio of 1.30 in OA and 1.14 in LA, respectively. *p* -Value of 0.795 indicated no statistically significant difference between the two groups with respect to gender. About 33.33% of LA were performed in acute appendicitis, 66.67% of LA were performed in recurrent appendicitis. Similarly, 30% of OA were performed in acute appendicitis, and 70% in recurrent appendicitis. *p* -Value >0.001 indicated no significant statistical difference.

**Fig. 4:**
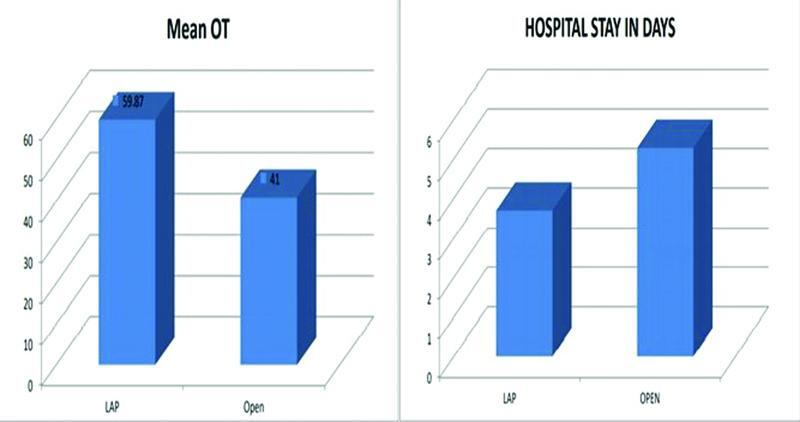
Graphs comparing mean operative duration and hospital stays in laparoscopic appendectomy (LA) and open appendectomy (OA). OT, operation theater.

The mean operative duration in LA and OA group was 47.17 ± 14.39 minutes and 36.9 ± 12.33 minute, respectively, with *p* = 0.001, indicating a statistically significant difference between the two groups with respect to operative times. This difference was attributable to the steep learning curve of laparoscopic procedure as no intraoperative complications were encountered in any of the LA prolonging the operative duration. The median VAS score in LA and OA group was 3.5 and 5, respectively, with *p* = 0.001, indicating significant statistical difference with respect to postoperative pain between the groups. The mean length of postoperative stay was 3.69 ± 0.71 days in LA group and 5.28 ± 0.63 days in OA group with a *p* = 0.000, indicating a significant statistical difference. The mean time to return to normal activity in LA group was 8.13 ± 1.33 days and that of OA group was 10.10 ± 2.20 days with *p* = 0.000, indicating a significant statistical difference. There were no conversions to open procedure in the LA group. No concomitant pathological findings were seen in both groups. Two cases (6.66%) in LA group and 4(13.33%) in OA group developed fever and serous discharge from the wound on the 2nd postoperative day, *p* = 0.652, indicating no significant statistical difference between the two groups with respect to postoperative wound-related complications. The mean scar scale scoring done on 30th postoperative day was 4.23 in LA and 8.23 in OA with *p* = 0.000, which suggested significantly better scars after LA in comparison to OA after 1 month of postoperative period ( Fig. 4 and Table 1 ).

**Table 1:** Demographic details and primary outcome measures

Parameter and outcome measures	LA ( *n* = 30)	OA ( *n* = 30)	*p* -Value
Median age (y)	25.2	24.9	0.221
Gender	Males—13 (43.3%)	Males—14 (46.7%)	
	Females—17 (56.7%)	Females—16 (53.3%)	
Female:male	1.14	1.30	0.795
Mean operative duration(min)	47.17 ± 14.39	36.9 ± 12.33	0.001
Median postoperative VAS score	3.5	5	0.001
Duration of postoperative hospital stays (d)	3.69 ± 0.71	5.28 ± 0.63	0.000
Mean time to return to normal activities (d)	8.13 ± 1.33	10.10 ± 2.20	0.000
Postoperative complications -Wound infection and fever	6.66%	13.33%	0.652
Mean scar scale score on 30th day	4.23	8.23	0.000

Abbreviations: LA, laparoscopic appendectomy; OA, open appendectomy; VAS, visual analogue scale.

## Discussion

“Appendix: forgettable, yet not so forgotten”

This underdeveloped residuum of the cecum has no known function and is commonly termed as a “vestigial” organ, yet diseases of the appendix loom large in surgical practice; and appendicitis continues to be the most common acute abdominal condition that requires immediate surgical treatment. 4 8 Early and prompt treatment will help in prevention of complications such as perforation, lump, and abscess formation. Though appendicitis presents with typical pain in the umbilical region at clinical presentation, which further localizes to right iliac fossa in 50 to 60% population, conditions such as ovarian cysts, ectopic pregnancy, pelvic inflammatory disease, and ileocecal Koch's are not uncommon differentials. 9 However, no evidence of negative appendectomies or misdiagnoses was found in our study. Both the groups in our study were comparable in terms of gender and ages of the patients with no significant statistical differences.

Consideration of operative duration in comparison of LA and OA has always been of importance in literature. The mean operative duration in OA was lesser than in LA in our study, which was statistically significant. This was in coherence with the studies by Yong et al with median operative duration being 80 minutes in LA and 60 minutes in OA groups and by Rashid et al with mean operative duration of 33.9 ± 78 minutes in OA group and 57.64 ± 9.89 minutes in LA group. 10 11 This was attributed to steeper learning curve of laparoscopic surgery. The median operative duration decreased with improving surgical skills of the surgeon over time in many studies. Both OA and LA were performed by a group of five surgeons in our study with adequate skills. However, as per the study by Khalil et al, the prolonged durations in LA can be attributed to additional maneuvers in LA such as creation of pneumoperitoneum, trocar insertion, and performing diagnostic laparoscopy that are absent in OA. 12 The mean duration of hospital stay in our study was 3.69 ± 0.71 days in LA group and 5.28 ± 0.63 days in OA group that was in corroboration to studies by Frazee RC et al, Malik et al, and Mulita et al. 13 14 15 Patients undergoing OA experienced more pain compared with the LA group that prolonged their recovery times and the duration of hospital stays. However, a study by Milewczyk et al showed no significant difference in postoperative hospital stays in LA group compared with the OA group. 16 Many authors have attributed the difference in postoperative hospital stays to the healthcare system rather than type of the procedure. 17 Kurtz and Heimann stated that the duration of hospital stay was determined by the appendiceal pathology rather than the type of procedure performed. 18 Patients with higher degrees of appendiceal inflammation were found to require longer hospital stays. Mean VAS scores in LA group were less than OA group. Increased pain in OA group was attributable to the length of fascial incision and stretch on the wound compared with the LA group as per a study by Kim et al. 19 A study by Rashid et al reflected similar findings with mean VAS score of 5.14 ± 0.132 in LA group and 6.01 ± 0.118 in the OA group. 11

Kehagias et al reported increased incidences of postoperative wound infections in OA group compared with LA group. 20 This was attributed to the delivery of infected appendix through the abdominal incision that increased the risk of infection. Safe delivery of the appendix in endobags is considered to reduce the chances of postoperative infection rates as stated by Aziz et al. 21 Surprisingly, the incidence of intraabdominal abscesses was found to be higher in LA group by Tang et al, that was attributed to the increased diffusion of infection due to high pressure in laparoscopy. 22 However, no statistically significant difference was seen in our study with respect to postoperative wound infections, which was also a finding in a study by Guller et al. 23 Return to normal activity depends on the country's culture and reimbursement systems. 24 However, the time taken after surgery in days when abdominal discomfort did not interfere with normal daily activities was considered in this study, which was significantly less in LA group. The patients in OA group took more time to return back to normal activities due to significant postoperative pain. Scar scale scoring performed at the 30th postoperative day revealed better scars in the LA group compared with the OA group pertaining to the length of the incisions. Multiple factors such as cost-effectiveness, stump appendicitis, and chronic complications were out of the scope of our study ( Table 2 ).

**Table 2:** Comparing the various parameters of different studies with the current study

Parameter →	Operative duration (min)		Postoperative VAS score		Duration of postoperative hospital stays (d)		Time to return to normal activities (d)		Postoperative complications	
Study ↓	LA	OA	LA	OA	LA	OA	LA	OA	LA	OA
Current study	47.17 ± 14.39	36.9 ± 12.33	3.5	5	3.69 ± 0.71	5.28 ± 0.63	8.13 ± 1.33	10.10 ± 2.20	6.66%	13.33%
Yong et al 10	80	60	–	–	2	3	–	–	13.4%	15.1%
Rashid et al 11	57.64 ± 9.89	33.9 ± 78	5.14 ± 0.132	6.01 ± 0.118	1.06 ± 0.2399	3.1 ± 0.8864	3.6 ± 1.03	9.64 ± 2.078	2%	2%
Frazee et al 13	87	65	–	–	–	–	14	25	–	–
Milewczyk et al 16	47.75	36.99	2.79	4.77	4.71	5.03	15.85	19.65	6.7%	9.4%
Kehagias I et al 20	44.3 ± 24	47 ± 19.7	–	–	–	–	–	–	8.1%	10.6%
Mulita et al 15	–	–	–	–	2.87	3.65	–	–	–	–

Abbreviations: LA, laparoscopic appendectomy; OA, open appendectomy; VAS, visual analogue scale.

## Conclusion

In conclusion, LA is more versatile approach than OA in the management of acute and recurrent appendicitis. Prolonged intraoperative duration was the only drawback with LA in our study; however, operative times were found to decrease with experience in literature. LA offered lesser operative site pain in the postoperative period, shorter postoperative hospital stays, leading to earlier recovery of the patient and return to normal activities. Cosmetically, LA was found to give better scars to the patient on a 30th day follow-up. Though wound-related complications were found to be higher in OA in literature, no significant difference was seen in our study.
